# Occupational cold exposure in relation to incident airway symptoms in northern Sweden: a prospective population-based study

**DOI:** 10.1007/s00420-022-01884-2

**Published:** 2022-05-31

**Authors:** Albin Stjernbrandt, Linnea Hedman, Ingrid Liljelind, Jens Wahlström

**Affiliations:** Section of Sustainable Health, Department of Public Health and Clinical Medicine, Umeå University, 901 87 Umeå, Sweden

**Keywords:** Asthma, Pulmonary disease, chronic obstructive, Cough, Cold exposure, Occupational exposure, Longitudinal studies

## Abstract

**Objective:**

To determine if occupational exposure to cold environments is associated with incident airway symptoms in previously healthy workers.

**Methods:**

A prospective, survey-based, closed-cohort study was conducted on a sample of 5017 men and women between 18 and 70 years of age, living in northern Sweden. Data on occupation, occupational and leisure-time cold exposure, airway symptoms, general health, and tobacco habits were collected during the winters of 2015 (baseline) and 2021 (follow-up). Stepwise multiple logistic regression was used to determine associations between baseline variables and incident airway symptoms.

**Results:**

For individuals working at baseline, without physician-diagnosed asthma or chronic obstructive pulmonary disease, reporting any occupational cold exposure was associated with incident wheeze (OR 1.41; 95% CI 1.06–1.87) and incident productive cough (OR 1.37; 95% CI 1.06–1.77), but not incident long-standing cough (OR 0.98; 95% CI 0.74–1.29), after adjusting for age, body mass index, daily smoking, and occupational physical workload. Detailed analysis of the occupational cold exposure rating did not reveal clear exposure–response patterns for any of the outcomes.

**Conclusions:**

Occupational cold exposure was robustly associated with incident wheeze and productive cough in previously healthy workers. This adds further support to the notion that cold air is harmful for the airways, and that a structured risk assessment regarding occupational cold exposure could be considered for inclusion in the Swedish workplace legislation. Further studies are needed to elaborate on exposure–response functions, as well as suggest thresholds for hazardous cold exposure.

**Supplementary Information:**

The online version contains supplementary material available at 10.1007/s00420-022-01884-2.

## Introduction

Living in a cold climate is related to a range of negative health effects (Mäkinen 2007). In the northern hemisphere, the winter season is associated with increased morbidity and mortality (Rocklöv and Forsberg 2008), especially among the elderly and those with preexisting cardiorespiratory disease (Näyhä 2005; The Eurowinter Group 1997). Having cold-related cardiorespiratory symptoms in turn predict a higher occurrence of hospital admissions and mortality (Ikäheimo et al. 2020). In more detailed analyses, larger effect sizes and more persistent effects have been described for cold-related respiratory diseases, compared to cardiovascular diseases (Analitis et al. 2008). In Scandinavia, almost a third of the general population report cold-related airway symptoms (Harju et al. 2010). Among individuals with asthma, a pronounced increase in obstructive airway symptoms have been shown both in epidemiological (Hyrkäs et al. 2014) and laboratory settings (Sjöström et al. 2019). In addition, an older study reported that more than one third of asthmatics avoid outdoor activities during the winter to prevent exacerbations (Millqvist et al. 1987). Similar deterioration has been described for subjects with chronic obstructive pulmonary disease (COPD), where cold spells have also been associated with increased airway symptoms and more frequent inhaler use (McCormack et al. 2017).

Apart from manifest diagnoses of asthma and COPD, several large epidemiological studies in Finland have indicated that airway symptoms, such as wheeze, cough, and increased sputum production are more prevalent in subjects living in cold climate (Kotaniemi et al. 2003; Näyhä et al. 2011; Raatikka et al. 2007), with an especially high occurrence among smoking outdoor workers (Kotaniemi et al. 2002). There are several indications that individuals *occupationally exposed* to cold climate are at higher risk for airway symptoms than other workers, which might be due to longer and more intense exposure to low temperatures, but also because of increased ventilation during strenuous manual work, inducing respiratory tract cooling and subsequent airway dysfunction (Mäkinen 2007; Stjernbrandt et al. 2021). Moreover, while some previous studies have reported that cold air is merely a trigger for airway symptoms (Koskela 2007), others have suggested that it might actually be a causal factor for the emergence of airway disease (Giesbrecht 1995; Kotaniemi et al. 2003). In 2015, we conducted a cross-sectional population-based study in northern Sweden, revealing associations between occupational cold exposure and reporting of airway symptoms (Stjernbrandt et al. 2021). However, there have been no previous longitudinal studies on the topic, and this paper represents a continuation of the previous project.

There are several ways to define cold climate exposure. One commonly used technical definition entails being subjected to ambient temperatures below 10 °C (International Organization for Standardization 2008). In settings where the temperature is not known, cold exposure has been defined as environmental factors that activate human thermoregulatory systems, or simply the subjectively reported experience of feeling cold (Ikäheimo 2011; Mäkinen et al. 2006). In the Nordic countries, the majority of inhabitants are daily exposed to ambient temperatures below 10 °C during the winter (The Swedish National Institute for Working Life 2002). According to official Swedish statistics, about 21% of men and 11% of women are occupationally exposed to cold climate for at least one quarter of the working hours, a pattern that been virtually unchanged during the last decade (The Swedish Work Environment Authority 2020). Finnish data suggest that workers with lower educational level and manual work tasks are more exposed to ambient cold, and that leisure-time cold exposure can be the dominant contribution (Mäkinen et al. 2006). However, the relation between occupational cold exposure and physical workload, as well as the interplay between occupational and leisure-time cold exposure, have not yet been fully understood.

The primary aim of the present study was to determine if occupational exposure to cold environments is associated with incident airway symptoms in previously healthy workers. Secondary aims were to explore the potential for an exposure–response pattern for occupational cold exposure, evaluate associations between occupational cold exposure and physical workload, and determine potential effects of leisure-time cold exposure on incident airway symptoms.

## Materials and methods

### Study design and setting

This longitudinal, population-based, closed-cohort study was a part of a research project titled Cold and Health In Northern Sweden (CHINS) that was launched in February of 2015 to investigate adverse health effects from cold climate exposure. Baseline data were collected during three months, by means of a postal survey on a sample of men and women between 18 and 70 years of age, living in northern Sweden, drawn from the national Swedish population register, as has previously been described in detail (Stjernbrandt et al. 2017). The survey collected information on occupation, cold exposure during work and leisure time, airway symptoms, general health, and tobacco habits. Responders of the baseline survey were asked again in 2021 to complete a similar but digital follow-up questionnaire during the late winter months. Responses from both surveys were subsequently merged based on the subject’s Swedish social security number.

### Outcome variables

Dichotomous dependent variables were: wheeze (“Have you at any time during the last 12 months had wheezing or whistling in your chest?”); long-standing cough (“Have you had long-standing cough during the last years?”); and productive cough (“Do you usually have phlegm when coughing, or do you have phlegm in your chest, which is difficult to bring up?”). Incident airway symptoms were defined as negating the dependent variable at baseline and giving a positive response at follow-up.

### Descriptive statistics and covariates

Occupation at baseline was reported in free-form text, and manually coded in accordance with the International Standard Classification of Occupations (ISCO) (International Labour Organization 2012). Physical workload was determined by a previously published job–exposure matrix (JEM) that categorized the exposure into low, medium or high, based on the two-level ISCO coding (Stjernbrandt and Hoftun Farbu 2022). Occupational or leisure-time cold exposure was assessed by two separate questionnaire items included in both surveys: “During work/leisure time I am exposed to outdoor or cold environments.” Answers were given on whole number numerical scales (NRS), ranging from one (“do not agree”) to ten (“fully agree”). Additional independent baseline variables used for adjusting were: age (years); body mass index (BMI; kg/m^2^); and daily smoking (yes/no). Cold exposure was subsequently categorized into four spans as well as dichotomized based on the 50th percentile, age groups into four similar spans, and BMI by clinically used thresholds for under- and overweight.

### Analyses

Since continuous variables were not normally distributed, data were described as median values and interquartile ranges (IQR), while categorical variables were presented as numbers and valid percentages (unless otherwise stated). The incidence proportion was calculated as the number of incident cases (as defined above) divided by subjects at risk (total sample minus subjects with symptoms at baseline), the remitted proportion as the number of remitted cases (having symptoms at baseline but not at follow-up) divided by the number of subjects with symptoms at baseline, and the persistent proportion as subjects with symptoms at baseline minus number of remitted cases, divided by the number of subjects with symptoms at baseline. Binary logistic regression was used for simple and multiple analyses. The multiple logistic regression was performed in a manual forward stepwise manner. Correlation between scales was investigated using Spearman’s rank correlation coefficient, and differences in proportions between groups with Pearson’s Chi-square test. A *p* value < 0.05 was considered statistically significant. Statistical analyses were performed using IBM SPSS Statistics for Windows (Version 27, IBM Corporation, Armonk, NY, USA).

## Results

### Recruitment

There were 12627 subjects responding to the baseline survey, of which 888 had died or moved from the study region during the follow-up time (2015–2021), leaving 11739 subjects eligible for follow-up. Out of these, 31 could not be reached by the postal services. There were 5208 responses to the follow-up survey, yielding a response rate of 44.4%. Due to multiple responses and invalid social security numbers, 191 survey responses could not be matched to the original dataset, which left 5017 subjects available for analysis. Five subjects (0.01%) opted to respond on paper, instead of digitally, for the follow-up survey.

### Descriptive data

Among responders to both surveys, the median (IQR) age was 60 (19) years at follow-up, and 2703 (53.9%) subjects were female. The majority (*N* = 2706; 53.9%) lived in the urbanized coastal region, 1209 (25.7%) in rural inland municipalities, and 1020 (20.3%) in sparsely populated alpine areas. Regarding baseline livelihood, 1093 (22.3%) were classified as professionals, 613 (12.5%) service and sales workers, 594 (12.1%) technicians and associate professionals, 475 (9.7%) clerical support workers, 290 (5.9%) plant and machine operators and assemblers, 259 (5.3%) managers, 239 (4.9%) crafts and related trades workers, 100 (2.0%) self-employed, 98 (2.0%) manual workers, 64 (1.3%) skilled agricultural, forestry, and fishery workers, and 18 (0.4%) professional militaries. In addition, 758 (15.4%) were retired, 154 (3.1%) students, 76 (1.5%) unemployed, 54 (1.1%) on sick leave, and 22 (0.4%) on parental leave.

Physician-diagnosed asthma at baseline was reported by 591 (12.0%) and COPD by 35 (0.7%). Other descriptive characteristics at baseline and follow-up are presented in Table 1. The prevalence**,** incidence, remission, and persistence proportions of airway symptoms are shown in Table 2.

**Table 1:** Descriptive characteristics of the study participants at baseline and follow-up

Variable	Categories	Baseline (2015)		Follow-up (2021)	
		*N*	%	*N*	%
Age group (years)	18–31	476	9.5	217	5.3
	32–44	872	17.4	670	16.3
	45–57	1603	32.0	1271	30.9
	58–70	2066	41.2	1953	47.5
Body mass index (kg/m^2^)	BMI < 20	190	3.9	183	3.8
	20 ≤ BMI < 25	2063	41.8	1885	38.7
	25 ≤ BMI < 30	1909	38.7	1933	39.7
	BMI > 30	769	15.6	864	17.8
Tobacco habits	Daily smoking	289	5.8	190	3.8
	Daily snuff use	665	13.4	686	13.8
Work status	Not working^a^	1064	21.7	1907	38.3
	Working	3843	78.3	3078	61.7
Occupational cold exposure	None (NRS 1)	2857	59.2	2190	51.0
	Any (NRS > 1)^b^	1969	40.8	2105	49.0
Leisure-time cold exposure	Low (NRS ≤ 5)	2442	49.5	1660	33.5
	High (NRS > 5)^b^	2487	50.5	3295	66.5
Airway symptoms^c^	Wheeze	936	18.7	837	16.7
	Long-standing cough	1086	21.7	772	15.4
	Productive cough	1000	19.9	942	18.8

*BMI* body mass index, *NRS* numerical rating scale

^a^Pensioners, students, unemployed, and those on sick or parental leave

^b^Dichotomized based the 50th percentile

^c^Affected by item non-response and presented as absolute percentages

**Table 2:** Prevalence, incidence, remitted, and persistent proportions of airway symptoms in the study population (*N* = 5017)

Measure	Wheeze^a^		Long-standing cough^a^		Productive cough^a^	
	*N*	%	*N*	%	*N*	%
Baseline prevalence	936/5017	18.7	1086/5017	21.7	1000/5017	19.9
Cumulative incidence proportion^b^	341/(5017–936)	8.4	350/(5017–1086)	8.9	417/(5017–1000)	10.4
Annual incidence proportion	341/(5017–936)/6	1.4	350/(5017–1086)/6	1.5	417/(5017–1000)/6	1.7
Remitted proportion^c^	442/936	47.2	658/1086	60.6	468/1000	46.8
Annual remitted proportion	442/936/6	7.9	658/1086/6	10.1	468/1000/6	7.8
Persistent proportion^d^	(936–442)/936	52.8	(1086–658)/1086	39.4	(1000−468)/1000	53.2

^a^The figures are affected by item non-response, and absolute percentages are presented

^b^The number of incident cases divided by subjects at risk (total sample minus subjects with symptoms at baseline)

^c^The number of remitted cases divided by the number of subjects with symptoms at baseline

^d^The number of persistent cases (subjects with symptoms at baseline minus number of remitted cases) divided by the number of subjects with symptoms at baseline

### Occupational cold exposure and physical workload

**Fig. 1:**
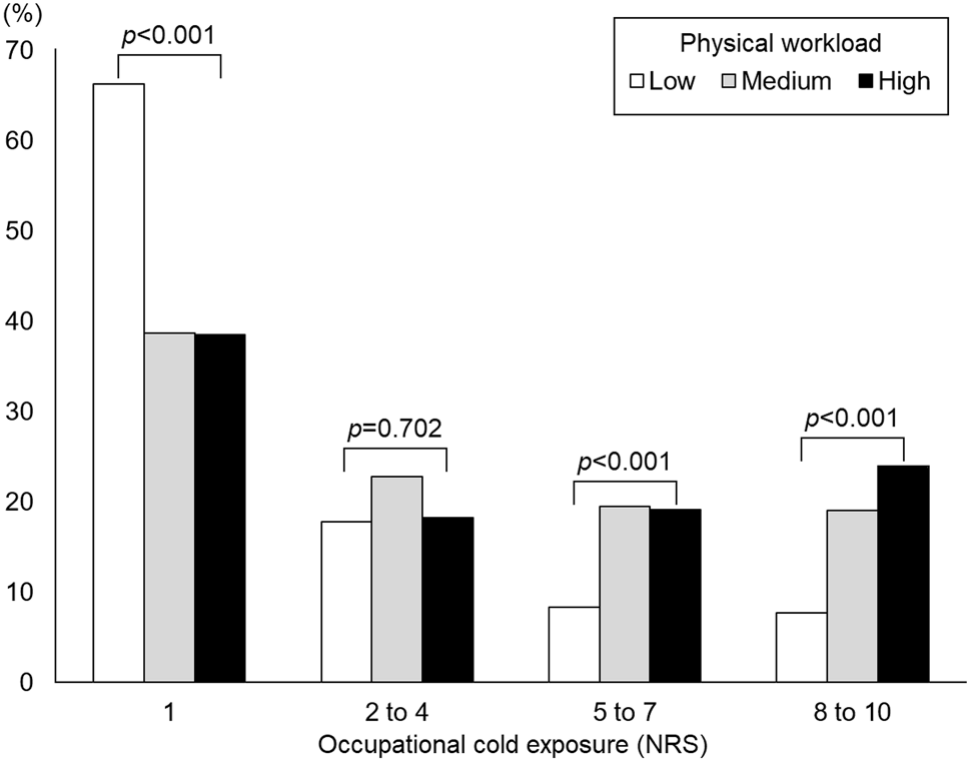
Proportions of subjects reporting different levels of occupational cold exposure at baseline, categorized by occupational physical workload, as determined by a three-level job-exposure matrix. *NRS* numerical rating scale

According to the JEM among subjects working at baseline, the physical workload was considered high in 779 (20.3%; e.g. climbing or heavy lifting), medium in 674 (17.5%; e.g. ambulatory tasks), and low in 2390 (62.2%; e.g. desk work). Spearman’s rank correlation coefficient between the ten-level occupational cold exposure scale and physical workload was 0.294 (*p* < 0.001). The proportions of occupational cold exposure, categorized by physical workload as determined by the JEM, are presented in Fig. 1.

### Cold exposure and incident airway symptoms

**Fig. 2:**
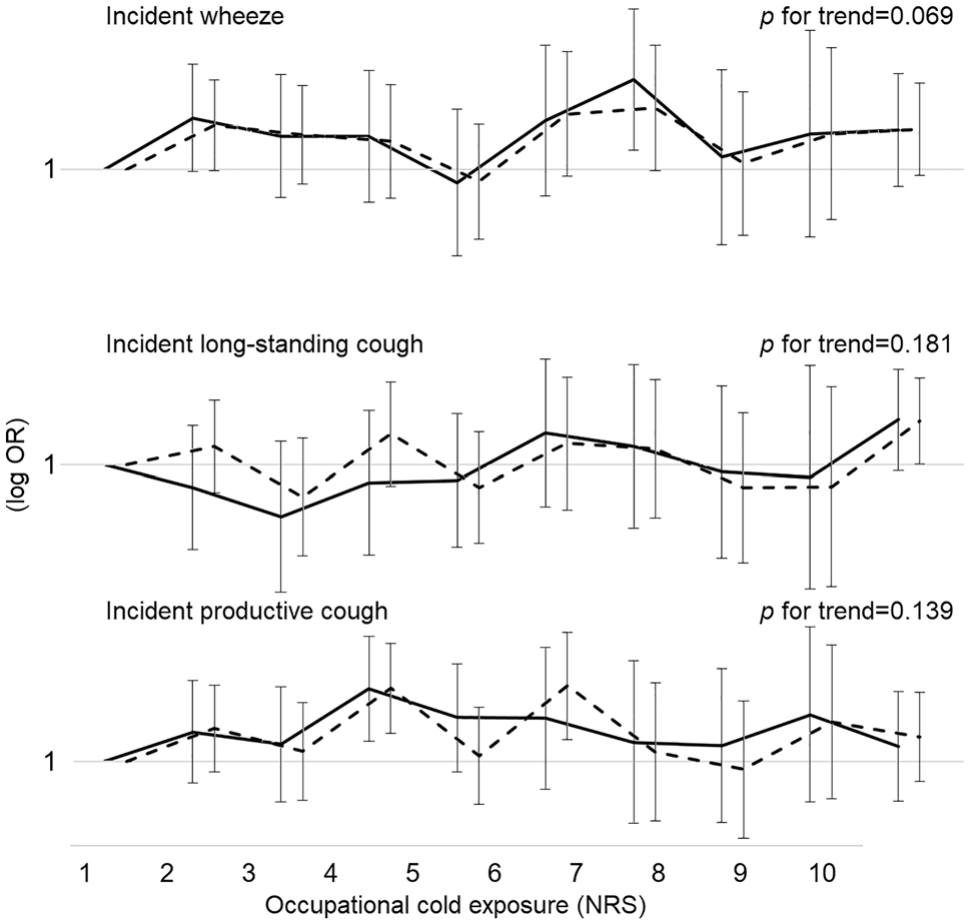
Exposure–response patterns showing crude (dashed lines) and adjusted odds ratios (solid lines) for reporting incident wheeze, long-standing cough, and productive cough on a logarithmic scale, in relation to occupational cold exposure, presented on a ten-level numerical rating scale. For the adjusted analyses, subjects not working at baseline, as well as those with physician-diagnosed asthma or chronic obstructive pulmonary disease have been excluded, and odds ratios have been adjusted for age, body mass index, daily smoking, and occupational physical workload. Error bars depict the ninety-five percent confidence intervals. Adjusted linear trend *p* values are also presented. *OR* odds ratio, *NRS* numerical rating scale

The crude OR (95% CI) for all subjects (*N* = 5017) between reporting any occupational cold exposure (above the 50th percentile, NRS > 1) and incident wheeze was 1.35 (1.08–1.69); for incident long-standing cough 1.08 (0.87–1.35); and for incident productive cough 1.32 (1.07–1.62). After excluding subjects with physician-diagnosed asthma (*N* = 591) and COPD (*N* = 35) at baseline, as well as those not working at baseline (pensioners, students, unemployed, and those on sick or parental leave; *N* = 1064), and adjusting for relevant baseline covariates (age, BMI, daily smoking, and occupational physical workload) the results were 1.41 (1.06–1.87); 0.98 (0.74–1.29); and 1.37 (1.06–1.77), respectively (Table 3). Detailed analysis of the occupational cold exposure rating did not reveal clear exposure–response patterns for any of the outcomes (Fig. 2). A full regression table for the final model is also available (Online Resource 1).

**Table 3:** Stepwise multiple logistic regression for the association between reporting any occupational cold exposure at baseline and incident wheeze, long-standing cough, or productive cough

Step	Any occupational cold exposure (NRS > 1) at baseline	Incident wheeze	Incident long-standing cough	Incident productive cough
		OR (95% CI)	OR (95% CI)	OR (95% CI)
I	Crude analysis	1.35 (1.08–1.69)*	1.08 (0.87–1.35)	1.32 (1.07–1.62)*
II	Physician-diagnosed asthma at baseline excluded (*N* = 591)	1.40 (1.10–1.79)*	1.03 (0.80–1.31)	1.32 (1.05–1.64)*
III	Physician-diagnosed chronic obstructive pulmonary disease at baseline excluded (*N* = 35)	1.42 (1.11–1.81)*	1.03 (0.80–1.31)	1.33 (1.06–1.66)*
IV	Non-workers at baseline excluded (*N* = 1064)	1.46 (1.11–1.93)*	0.99 (0.75–1.29)	1.34 (1.04–1.72)*
V	Adjusted for age at baseline (continuous)	1.47 (1.12–1.94)*	0.99 (0.75–1.29)	1.37 (1.06–1.75)*
VI	Adjusted for body mass index at baseline (continuous)	1.40 (1.06–1.85)*	0.96 (0.73–1.27)	1.36 (1.06–1.74)*
VII	Adjusted for daily smoking at baseline (yes/no)	1.38 (1.04–1.82)*	0.96 (0.73–1.26)	1.36 (1.05–1.74)*
VIII	Adjusted for occupational physical workload at baseline (high/low to medium)	1.41 (1.06–1.87)*	0.98 (0.74–1.29)	1.37 (1.06–1.77)*

*OR* odds ratio, *NRS* numerical rating scale, *95% CI* ninety-five percent confidence interval

*Significant at the 0.05 level

The corresponding crude OR (95% CI) for all subjects (*N* = 5017) between reporting leisure-time cold exposure (above the 50th percentile, NRS > 5) and incident wheeze was 0.95 (0.76–1.19); for incident long-standing cough 0.98 (0.79–1.23); and for productive cough 1.00 (0.82–1.23). After excluding subjects with asthma and COPD at baseline, and adjusting for age, BMI, and daily smoking, the results were 0.96 (0.76–1.23); 0.99 (0.78–1.26); and 1.02 (0.82–1.26), respectively.

## Discussion

### Main results

This prospective survey with a follow-up time of six years, conducted on a sample of the working general population in northern Sweden, found that occupational cold exposure at baseline was associated with incident wheeze and incident productive cough, but not incident long-standing cough, in an adjusted logistic regression model. There were no clear exposure–response patterns for occupational cold exposure in relation to the outcomes. An association between occupational cold exposure and physical workload was found, where many of those with the highest exposure to cold also reported a high physical workload. The annual incidence proportion was lower than the remission proportion, resulting in a decreasing prevalence of airway symptoms over time. Leisure-time cold exposure was not significantly associated with incident airway symptoms.

### Interpretation

The main results of the present study are in line with previous population-based studies, showing increased airway morbidity from cold air exposure (Analitis et al. 2008; Hajat et al. 2007; Harju et al. 2010; Kotaniemi et al. 2002; Millqvist et al. 1987; Näyhä 2005; Schwartz 2005; The Eurowinter Group 1997). The effect size was roughly comparable with a cross-sectional Finnish study reporting associations between occupational cold exposure and shortness of breath (OR 1.23; 95% CI 1.03–1.47) and chronic bronchitis (OR 1.77; 95% CI 1.21–2.60) (Kotaniemi et al. 2003). However, most of the previous literature consists of either cross-sectional surveys or ecological time series on aggregated data, and many do not focus on occupational exposure. Thus, to the authors’ knowledge, there are no previous longitudinal studies of similar design to compare with. The prevalence of self-reported physician-diagnosed asthma in the present study was roughly 12%, which was somewhat higher than other recent Scandinavian studies, reporting figures between 9 and 11%, depending on region and age distribution of the study samples (Backman et al. 2017; Harju et al. 2010). For COPD, the prevalence in the present study was about 1%, which was notably lower than the range of 3–4% reported by other authors (Backman et al. 2016; Harju et al. 2010; Kotaniemi et al. 2002). This range in prevalence can be attributed to differences in case definitions, smoking habits, age distribution, and sampling. Also, studies basing diagnoses on lung function tests are likely to be more sensitive than those only using survey responses, since spirometry can reveal mild obstructive lung disease in asymptomatic individuals (Lindberg et al. 2006). In the present study, smoking declined from roughly 6 to 4% during the study period, both figures substantially lower than Swedish data from 2009 to 2017, indicating habitual smoking in 10–12% of the general population (Backman et al. 2017; Eriksson et al. 2011). This difference might reflect an ongoing decrease in smoking over time, but possibly also an effect of the overrepresentation of subjects living in the coastal region in the present study, where socio-economic status is higher and smoking less common (Rodu et al. 2013).

As seen in Table 1, the occurrence of wheeze, long-standing cough and productive cough was somewhat higher at baseline (19–22%) compared to follow-up (15–19%). This is line with the findings in Table 2, where the annual incidence proportion was lower than the remitted proportion. This decline in airway symptoms over time has been reported by other authors (Backman et al. 2014), and the explanation is likely multifactorial. Most obviously, the change might be related to the diminishing smoking trend in Sweden, that has been reported in large surveys (Backman et al. 2014; Stegmayr et al. 2005), and was also found in the present study. It might also be related to more effective inhaler treatment of obstructive lung diseases (Ekerljung et al. 2014). In contrast, the change is not likely to be due to a general decrease in exposure to cold work environments, and the present study rather indicates an increase in occupational cold exposure between baseline and follow-up (Table 1). Finally, concerns can be raised about potential selection bias in the present study affecting the prevalence estimates, where subjects with emerging airway symptoms during the study period might be less likely to remain in the workforce, and respond to a follow-up questionnaire on occupational health.

In the first cross-sectional study on this cohort, both wheeze, long-standing cough and productive cough were associated with occupational cold exposure (Stjernbrandt et al. 2021). However, in this longitudinal continuation, associations were found only for incident wheeze and productive cough, but not incident long-standing cough. In the previous literature, wheeze has been described to indicate airway obstruction that is present in adults with both asthma and COPD, while long-standing and productive cough are more strongly related to chronic bronchitis as a feature of COPD (Harju et al. 2010; Martin and Harrison 2015). In addition, productive cough is also associated with airway infections, to which the susceptibility increases with cold exposure (Eccles 2002; Mäkinen and Hassi 2009). Thus, the lack of association between cold exposure and long-standing cough in the present study can be interpreted as support for the notion that ambient cold does not increase the risk of incident chronic bronchitis. However, there is also a possibility that the results are affected by the length of latency periods, i.e. that asthmatic symptoms develop rather rapidly, while chronic cough would mandate a longer follow-up time for an association to become discernible. The present study could not conclude on a convincing exposure–response pattern for occupational cold exposure. It is possible, although not plausible, that the magnitude of exposure is irrelevant above a certain threshold. More likely, the exposure measure in the present study was simply not detailed enough to capture such effects. There were no clear effects of leisure-time cold exposure on incident airway symptoms, well in line with the previous study (Stjernbrandt et al. 2021). It has not been fully understood why occupational cold exposure appears to be more strongly associated with airway symptoms than leisure-time exposure, even though the latter can constitute a larger contribution to the total exposure on a population level (Mäkinen et al. 2006). Possible explanations could be that occupational exposure is generally of longer duration, demands a higher physical activity level, and cannot easily be refrained from during intensely cold weather conditions.

From a technical standpoint, the hazardous potential of occupational cold exposure is likely dependent on ambient temperature, air humidity level, wind speed, and exposure duration. Individual factors, such as body composition and clothing subsequently modify the effects of cold exposure. One additional important individual factor is the degree of physical strain, which is generally considered to attenuate the effects of cold exposure by increasing heat production. However, in the context of respiratory tract symptoms, the opposite may be true, since strenuous work induces a higher respiratory rate, and a shift from nose to mouth breathing, which increases the exposure of the airways to cold and dry air (Koskela 2007; McFadden et al. 1985). Apart from health concerns, cold-induced respiratory problems can also negatively affect the work performance, which is important from an employer perspective (Koskela et al. 1998; Mäkinen and Hassi 2009). Since the present study adds to the growing body of data showing adverse health effects from occupational cold exposure, benefits in both health and productivity could likely be gained from updating the national workplace legislation, mandating a structured risk assessment of cold work tasks, and periodical medical examinations for highly exposed occupational groups, based on the current international standard for risk assessment and management of cold workplaces (International Organization for Standardization 2008).

The present study included an adjusted regression model, with several restrictions and covariates. First of all, only currently working subjects were included, since the focus was on occupational cold exposure. Moreover, as the purpose was to find incident airway disease, subjects with physician-diagnosed asthma and COPD at baseline were excluded. Since previous studies have indicated that cold-related respiratory symptoms are more common in smokers (Harju et al. 2010; Kotaniemi et al. 2002) and increase with age (Kotaniemi et al. 2002; Mäkinen and Hassi 2009), both daily smoking and age were included as covariates. BMI was included as an indicator of general health. The inclusion of occupational physical workload was motivated by the fact that high physical demands induce a higher ventilatory rate which in turn increases the cold exposure in the airways (Koskela 2007). Restricting and adjusting the model, using all these factors, only had a minor impact on the effect size, indicating that occupational cold exposure was indeed robustly associated with incident airway symptoms.

### Limitations

One of the major potential limitations with the present study was the rather low response rate, which may have introduced a sampling bias regarding both exposure and outcome variables. Other possible limitations include the self-reported cold exposure variables that have not been validated against other means of exposure measurements, and the use of a JEM which was solely based on occupational title, to offer some perspective of the overall physical workload. The survey items used for cold exposure assessment were arbitrarily scaled, and cannot easily be converted into exposure intensity (e.g. temperature) or duration (e.g. hours per day). Furthermore, there was a large number of subjects that were not working at baseline (*N* = 1064; 21.7%), and this proportion increased over the course of the study, possibly attenuating the occupational perspective. Finally, the study did not investigate other acknowledged occupational risk factors for airway symptoms, such as exposure to gases, vapors, fumes, dusts, and mists (Omland et al. 2014; Schyllert et al. 2016).

### Strengths

To the authors’ knowledge, this is the first prospective study on occupational cold-related airway symptoms in this setting, and it included more than five thousand subjects, taking on the perspective of the general population. The surveys collected ample background data to enable an adjusted regression model. There is no reason to believe that the findings should not be representative of the general population in northern Sweden as a whole, since the prevalence estimates closely resemble those of other recent Swedish studies (Backman et al. 2016, 2017; Eriksson et al. 2011). Since both data collection efforts were performed during the same late winter period, the effects of cold exposure would not be expected to differ between baseline and follow-up.

### Conclusions

Occupational cold exposure was robustly associated with incident wheeze and productive cough in previously healthy workers. This adds further support to the notion that cold air is harmful for the airways, and that a structured risk assessment regarding occupational cold exposure could be considered for inclusion in the Swedish workplace legislation. Detailed analysis of the occupational cold exposure rating did not reveal clear exposure–response patterns for any of the outcomes. Further studies are needed to elaborate on exposure–response functions, as well as suggest thresholds for hazardous cold exposure. There was an association between occupational cold exposure and physical workload, and this likely affects the respiratory rate and pattern, modifying the cold exposure that the airways are subjected to. Finally, leisure-time cold exposure was not related to any of the outcomes, and should therefore not be focused on when planning preventive measures.

## Supplementary Information

Below is the link to the electronic supplementary material.

Supplementary file1 (PDF 86 KB)

## Data Availability

Source data can be made available upon personal request.
