# Supplementary material for: Occupational cold exposure in relation to incident airway symptoms in northern Sweden: a prospective population-based study
Source: Int Arch Occup Environ Health. 2022 May 31;95(9):1871–9. doi: 10.1007/s00420-022-01884-2 (PMC9630183; doi:10.1007/s00420-022-01884-2)
Supplement: Supplementary file 1 — Supplementary file1 (PDF 86 KB) [file 420_2022_1884_MOESM1_ESM.pdf]

Article title: Occupational cold exposure in relation to incident airway symptoms in northern Sweden – a prospective population-based study

Journal name: International Archives of Occupational and Environmental Health

Author names: Albin Stjernbrandt[1]; Linnea Hedman[1]; Ingrid Liljelind[1]; Jens Wahlström[1]

Affiliations: [1]Section of Sustainable Health, Department of Public Health and Clinical Medicine, Umeå University

E-mail address of the corresponding author: albin.stjernbrandt@umu.se

Online Resource 1 Odds ratios and ninety-five percent confidence intervals from simple logistic regression analyses and the final multiple model, for reporting incident wheeze, long-standing cough, or productive cough

		**Incident wheeze**		**Incident long-standing cough**		**Incident productive cough**	
		**Simple**	**Multiple** [a]	**Simple**	**Multiple** [a]	**Simple**	**Multiple** [a]
**Baseline variable**	**Categories**	OR (95% CI)	OR (95% CI)	OR (95% CI)	OR (95% CI)	OR (95% CI)	OR (95% CI)
Occupational cold exposure	None (NRS≤1)	1.00	1.00	1.00	1.00	1.00	1.00
	Any (NRS 2–10)	1.35 (1.08–1.69)*	1.41 (1.06–1.87)*	1.08 (0.87–1.35)	0.98 (0.74–1.29)	1.32 (1.07–1.62)*	1.37 (1.06–1.77)*
Age	Years	1.00 (0.99–1.01)	1.00 (0.99–1.01)	1.00 (0.99–1.00)	1.00 (0.98–1.01)	1.01 (1.00–1.02)*	1.01 (1.00–1.03)*
Body mass index	Kg/m²	1.05 (1.03–1.08)*	1.07 (1.04–1.09)*	1.02 (0.99–1.04)	1.03 (1.00–1.06)*	1.02 (1.00–1.04)*	1.02 (0.99–1.05)
Daily smoking	No	1.00	1.00	1.00	1.00	1.00	1.00
	Yes	1.64 (1.11–2.44)*	2.12 (1.34–3.37)*	1.52 (1.02–2.27)*	2.07 (1.31–3.27)*	1.51 (1.04–2.19)*	1.40 (0.88–2.23)
Occupational physical workload	Low or medium	1.00	1.00	1.00	1.00	1.00	1.00
	High	0.93 (0.68–1.27)	0.84 (0.58–1.19)	0.88 (0.65–1.20)	0.83 (0.58–1.19)	0.91 (0.68–1.22)	0.91 (0.66–1.25)

OR odds ratio, *NRS* numerical rating scale, *95% CI* ninety-five percent confidence interval

*Significant at the 0.05 level

[a] Excluding subjects with physician-diagnosed asthma (N=591) and COPD (N=35) at baseline, as well as those not working at baseline (pensioners, students, unemployed, and those on sick or parental leave; N=1,064), and adjusted for all other covariates in the table
